# Paradigms about the COVID-19 pandemic: knowledge, attitudes and practices from medical students

**DOI:** 10.1186/s12909-021-02559-1

**Published:** 2021-02-24

**Authors:** Eddy Lincango-Naranjo, Nataly Espinoza-Suarez, Paola Solis-Pazmino, Paul Vinueza-Moreano, Santiago Rodriguez-Villafuerte, Jose Lincango-Naranjo, Giuseppe Barberis-Barcia, Carlos Ruiz-Sosa, Giovanni Rojas-Velasco, Derek Gravholt, Elizabeth Golembiewski, Percy Soto-Becerra, Maryam Khan, Esteban Ortiz-Prado

**Affiliations:** 1 Knowledge and Evaluation Research Unit, Mayo Clinic, Rochester, Minnesota USA; 2 Facultad de Ciencias Médicas, Universidad Central del Ecuador, Quito, Ecuador; 3 Department of Otolaryngology-Head and Neck Surgery, School of Medicine, Stanford University, Stanford, CA USA; 4 Facultad de Medicina, Programa de Pós-graduação em Hepatología, Universidade Federal de Ciências da Saúde de Porto Alegre (UFCSPA), Porto Alegre, RS Brazil; 5 Hospital Vozandes Quito, Quito, Ecuador; 6 Universidad de las Fuerzas Armadas, Quito, Ecuador; 7 Universidad de Sao Paulo, Sao Paulo, Brazil; 8 Centro de Excelencia en Investigaciones Económicas y Sociales de Salud, Universidad San Ignacio de Loyola, Lima, Peru; 9 University Hospital Southampton NHS Foundation Trust, Southampton, UK; 10 OneHealth Global Research Group, Universidad de las Americas, Ecuador Calle de los Colimes y Avenida De los Granados, 170137 Quito, Ecuador

**Keywords:** SARS-CoV-2, Medical students, Attitudes, Perception, Knowledge, COVID-19

## Abstract

**Background:**

As the disease caused by the novel coronavirus has spread globally, there has been significant economic instability in the healthcare systems. This reality was especially accentuated in Ecuador where, the shortage of healthcare workers combined with cultural and macroeconomic factors has led Ecuador to face the most aggressive outbreak in Latin America. In this context, the participation of final-year medical students on the front line is indispensable. Appropriate training on COVID-19 is an urgent requirement that universities and health systems must guarantee. We aimed to describe the knowledge, attitudes, and practices of Ecuadorian final-year medical students that could potentially guide the design of better medical education curricula regarding COVID-19.

**Methods:**

This was a cross-sectional 33-item online survey conducted between April 6 to April 2020 assessing the knowledge, attitudes, and practices toward the diagnosis, treatment, prevention, and prognosis toward COVID-19 in Ecuadorian final-year medical students. It was sent by email, Facebook, and WhatsApp.

**Results:**

A total of 309 students responded to the survey. Out of which 88% of students scored high (≥ 70% correct) for knowledge of the disease. The majority of students were pessimistic about possible government actions, which is reflected in the negative attitude towards the control of COVID-19 and volunteering during the outbreak in Ecuador (77%, and 58% of the students, respectively). Moreover, 91% of students said they did not have adequate protective equipment. The latter finding was significantly associated with negative attitudes.

**Conclusions:**

Although a large number of students displayed negative attitudes, the non-depreciable percentage of students who were willing to volunteer and the coexisting high level of knowledge displayed by students, suggests that Ecuador has a capable upcoming workforce that could benefit from an opportunity to strengthen, improve and advance their training in preparation for COVID-19. Not having personal protective equipment was significantly associated to negative attitudes. Providing the necessary tools and creating a national curriculum may be one of the most effective ways to ensure all students are trained, whilst simultaneously focusing on the students’ most pressing concerns. With this additional training, negative attitudes will improve and students will be better qualified.

**Supplementary Information:**

The online version contains supplementary material available at 10.1186/s12909-021-02559-1.

## Introduction

The severe acute respiratory syndrome coronavirus-2 (SARS-CoV-2) is a zoonotic respiratory virus that causes the novel coronavirus disease-2019 (COVID-19) [1]. Due to its high infectivity and airborne-based transmissibility, SARS-CoV-2 has quickly spread globally, infecting more than 40 million and killing more than 1.2 million people worldwide [2]. As far as we know, the first cases of this novel-respiratory infection were reported in China in November 2019, was sequenced on January 7th, and the pandemic was declared on March 11th [3].

At the beginning of the outbreak, very few countries including Italy, Spain and Ecuador experienced the most abrupt COVID-19 surge in the number of new cases, exerting unprecedented pressure on their health systems [4]. In Latin America, the arrival of the virus is worrisome. This region has one of the highest urbanization rates in the world, a region with substantial socioeconomic inequities and debilitated health systems. On February 27th, 2020 Ecuador reported its first official case of COVID-19; one month later the coastal region of the country faced the most aggressive COVID-19 outbreak, reporting the highest excess mortality per-capita in the world [5]. In this low-middle income country, strict control measures, including limiting international flights, social distancing, wearing face masks, and closing nonessential businesses, and declaration of emergency took place earlier than their neighbors; nevertheless, these interventions alone have not been successful in stopping the spread of COVID-19 and the number of cases has continued to increase [6].

The struggle to provide acute health coverage to the unprecedented amount of diseased, put healthcare workers (HCWs) to the limit. Several hospitals reported a shortage of medical personnel, forcing authorities to call upon medical students to assist in the already overwhelmed national health system [7].

During the last year of medical school, Ecuadorian medical students are a critical piece for the system and are immersed in a range of key clinical activities within the health system. Their role has developed from merely observing, to being actively involved in patient care responsibilities. They belong to the novel generations of healthcare practitioners. Although they have little experience managing patients or using personal protective equipment properly, they have been allocated in several clinical wards in order to tackle the COVID-19 crisis.

During the initial months of the pandemic, healthcare workers including medical students were exposed to the SARS-CoV-2 virus without proper protective equipment [8]. Around the world, a large number of HCWs have been infected with COVID-19 and thousands have died [9–11]. In Ecuador alone, 1600 HCWs have tested positive for the virus and 50 HCWs have died since the begging of the pandemic [4].

At present, no treatment is available to prevent COVID-19, leaving the application of preventive measures as the most critical intervention available [12]. Having positive attitudes towards these guidelines and adhering to the practice of these measures could be essential to avoid deaths and facilitate outbreak management. In our study, we aim to describe the knowledge, attitudes, and practices towards the diagnosis, treatment, prevention, and prognosis of COVID-19, among Ecuadorian final-year medical students. We believe this information could potentially guide the design of better medical education curricula to approach acute health emergencies such as COVID-19.

## Methods

### Setting and participants

A cross-sectional, online survey was fielded from April 6 to April 20, 2020, during the first phase of the pandemic, a critical period to evaluate how prepared medical students were to respond to this public health emergency. Participants were students currently enrolled in their final year of medical school in Ecuador. According to the secretary of the Association of Ecuadorian Faculties of Medical and Health Sciences (AFEME, in Spanish) the estimated whole population of final-year medical students in Ecuador was 3000 [13]. Base on this population and assuming a response proportion of 50%, a 95% confidence level and a 5% margin error, a sample size of 379 respondents was calculated using App4Stats [14]. The secretary sent the survey by email to the deans of all 22 universities with a medical school in Ecuador for distribution to all final year medical students at each school. The survey was also sent to eligible participants by the co-authors’ through Facebook and WhatsApp groups related to final-year medical students, using snowballing as a secondary sample method (P.V-M., C.R-S.,  G.B-B.). The participants were encouraged to roll out the survey among other medical students. Weekly reminders were sent to medical students and deans during the time frame of the survey.

### Survey development and measures

The data was collected using a 33-item online survey to evaluate medical students’ knowledge, attitudes, and practices (KAP) around COVID-19. A novel survey instrument was developed for this study using items adapted from information about COVID-19, published by the CDC and the WHO alongside items used in previous COVID-19 surveys [12, 15, 16]. The questionnaire consisted of four sections: demographic characteristics, COVID-19 related knowledge, attitudes and practices. The survey was developed and fielded in Spanish and later translated into English for reporting purposes. The survey was conducted using Google Forms, an online, cloud-based survey administration application. To minimize missing data, respondents were required to complete each item to proceed to the subsequent items. The full survey instrument is available in the Additional file 1.

The questionnaire was reviewed for face validity by three experts in medical education, infectious diseases, and biostatistics to identify key issues that may be relevant to final-year medical students during the outbreak and to assess its relevance and accuracy. After incorporating expert feedback, we pilot-tested the survey instrument online with a group of 30 final-year medical students. During this process, students completed the survey in full and then were interviewed by three members (E.L-N., P.V-M., C.R-S.) of the research team to elicit their feedback and suggestions for improvement. The 30 students who completed the pilot-testing did not participate in the final survey and the responses collected during pilot-testing were not included in the final analysis.

### Demographic characteristics

We collected demographic information from respondents including; age, sex, name, and location of their university and the current hospital and department where students were being trained.

### Domain: knowledge

The knowledge domain was composed of 23 questions. The questions evaluated students’ knowledge about COVID-19 including its; virology, diagnosis, clinical management, prevention, and relevant infection control measures. For all questions in this domain, respondents were asked to respond ‘true’, ‘false’ or ‘not sure’. A correct answer was assigned 1 point and an incorrect answer or a ‘not sure’ response was assigned 0 points. For each respondent, we categorized a knowledge score of ≥16 points out of 23 possible points (≥ 70% correct) as “high knowledge”. Scores of < 16 points (< 70% correct) were categorized as “low knowledge”. The cut-off point is based on the academic approval regulations of the Universidad Central del Ecuador, which considers a demanding cut-off point of 70% of the final grade, which is the minimum necessary to consider a high level of knowledge.

### Domain: attitudes

In the attitude domain, students were asked four questions about their opinions toward volunteering at a health facility during the COVID-19 outbreak and disease control in hospitals in Ecuador. All questions in the attitude domain had “yes” or “no” response options.

### Domain: practices

Finally, the practices domain was composed of six questions. This domain evaluated the students’ use of guidelines, training, conferences, hand washing, and scientific searches for information (including types of sources consulted). Four questions that required a ‘yes’ or ‘no’ response and two single-select multiple-choice questions.

### Data management

Demographic characteristics data were managed as follows: universities were classified as public and private according to the secretariat of higher education science and technology (Senescyt) [17] from Ecuador. The hospitals were grouped as public and private subsystems. The public system is comprised of facilities run by the Ministry of Public Health, the Ecuadorian Social Security Institute (which includes Rural Social Security, the Armed Forces, and the National Police), and the health services of some municipalities. The private system is comprised of health insurance companies and prepaid plans for medicine providers [18]. The rotation department was classified into clinics, surgical, gynaecology, or paediatrics. The cities were grouped depending on the sales income reported in 2018 (in billions of dollars) according to INEC: Quito (more than 50 billion), Guayaquil (between 50 billion and 10 billion), and others (less than 10 billion) [19].

### Statistical methods

Descriptive statistics were computed using numbers and percentages for all categorical variables and median and interquartile ranges for all numerical values. The normality of each numerical variable was evaluated with the Kolmogorov Smirnov test. Our dependent variables used for exploratory analysis were attitude questions. Chi-square test and multivariable binary logistic regression were used to estimate respondent demographic factors, knowledge and practices associated with greater odds of not being willing to volunteer at a health facility during the COVID-19 crisis, perception that the respondent’s health facility is not prepared for a COVID-19 outbreak, and perception that Ecuador will not be able to control the COVID-19 outbreak. Factors were selected with the enter method. The results are reported as odds ratios (OR) and with their respective 95% confidence intervals. The data was analysed using IBM-SPSS version 19 (IBM, Armonk, NY, USA), and *P* < 0·05 was considered statistically significant.

## Results

A total of 309 final-year medical students completed to the survey, yielding a response rate of 81.5%. Descriptive characteristics of the study sample are reported in Table 1. A majority of participants were women (56.3%), between 23 and 25 years old (74.1%), studied in Quito (61.4%), and in a public university (67.9%). Nearly all (94.2%) students were currently training in a public hospital.

**Table 1:** Demographic characteristics

Total participant (***n*** = 309)	n	(%)
**Age (median: 24, IQ: 16–19)**		
Sex		
Female	174	(56.3%)
Male	135	(43.7%)
**Study city**		
Quito	190	(61.4%)
Guayaquil	19	(6.14%)
Others^a^	100	(32.5%)
**University**		
Public	210	(67.9%)
Private	99	(32.1%)
**Hospital type**		
Public	291	(94.2%)
Private	18	(5.82%)
**Department**		
Surgery	38	(12.3%)
Pediatry	51	(16.5%)
Gynecology	54	(17.4%)
Clinic	166	(53.8%)

^a^ Other cities included the following respondents (n): Cuenca: 34, Ambato 23; Manta: 18; Esmeraldas: 8; Tulcan: 5; Ibarra: 4; Riobamba: 3; Latacunga: 1; Loja: 1; Machala: 1; Quevedo: 1; Santa Elena: 1

### Knowledge about COVID-19

Across the entire study sample, the median COVID-19 knowledge score was 17 points out of a possible 23 points (IQR: 16, 19, range: 12–22). The vast majority of participants (88.0%) attained a high knowledge score (≥16). Out of the 309 respondents, 8.0% scored 20–23 of 23 possible knowledge points, 80.6% scored 16–19 points, and 11.4% scored 12–15 points. The lowest scores were obtained in the knowledge of the subgenus to which the virus belonged (31.4%) and the basic structure of the virus (68.6%). Other questions are detailed in Table 2.

**Table 2:** Descriptive statistics for survey responses

Total participants (***n*** = 309)		Correct, n	(%)
**Knowledge domain**			
**General knowledge**			
1. Is SARS-CoV-2 a new coronavirus identified at the end of 2019, and when it infects humans, causes acute respiratory infection?		298	(96.4)
2. Is SARS-CoV-2 a positive-sense single-stranded RNA virus?		207	(67.0)
3. Is SARS-CoV-2 a member of the subgenus Sarbecovirus (beta-CoV B lineage)?		97	(31.4)
4. Can SARS-CoV-2 live for some time on some surfaces?		306	(99.0)
5. Not everyone with COVID-2019 will develop serious cases. Are people who have the following characteristics more likely to have serious cases?: age over 65 years, have comorbidities, obese.		302	(97.7)
6. Would being in contact with your pets lead to SARS-CoV-2 virus infection?		304	(98.4)
7. Do you think SARS-CoV 2 stays in the air for 3 h?		143	(46.3)
**Diagnosis**			
8. Are the most prevalent symptoms of COVID-19 fever, cough and fatigue?		303	(98.1)
9. Is the main incubation period for COVID-19 1–30 days?		225	(72.8)
10. Is the main transmission mechanism of SARS-CoV-2 close person-to-person contact between people infected with the virus, whether symptomatic or asymptomatic?		284	(91.9)
11. Is the diagnosis for COVID-19 recommended by the WHO made by a rapid test?		96	(31.1)
12. Is the diagnosis for COVID-19 recommended by the WHO made by the polymerase chain reaction (RT-PCR) test of the nasopharyngeal swab?		299	(96.8)
13. Is a suspicious case of COVID-19 defined as any patient who meets the clinical picture of acute respiratory syndrome (fever, cough, dyspnea, fatigue), and / or epidemiological criteria (being in contact with a suspected or confirmed case of COVID 19, travel or residence in area with active infections in the last 14 days), but without confirmation by laboratory test?.		300	(97.1)
14. Is a confirmed case of COVID-19 defined as any patient who meets the clinical picture of acute respiratory syndrome and / or epidemiological criteria confirmed by laboratory testing?		293	(94.8)
**Treatment**			
15. There is currently no effective cure for COVID-2019, but does early supportive and symptomatic treatment help most patients recover from infection?		300	(97.1)
16. Is the use of chloroquine is recommended as prophylaxis for COVID-2019?		274	(88.7)
**Prevention**			
17. Should hand hygiene be performed for more than 20 s, mainly hand washing with soap?		279	(90.3)
18. Does the personal protective equipment recommended by the WHO, for the care of a suspected or confirmed case of COVID-19, without aerosol-generating procedures, include: hand hygiene, N95 mask, gloves, gown, and protective glasses?		30	(9.7)
19. Was the recommendation of distance between patients and health personnel as far as possible due to the fact that the macroparticles generated by coughs or sneezes spread up to 2 m away, and therefore are potential virus transporters?		279	(89.0)
20. Do you think that the life time of the N95 mask is 7 days?		55	(17.8)
21. Is Isolation an effective way to reduce the spread of the virus?		304	(98.4)
**Prognosis**			
22. Can patients affected by COVID-19 recover from the disease?		304	(98.4)
23. Can people recovered from the disease still transfer or spread it?		192	(62.2)
**Total knowledge score, n (%)**			
≥ 16 correct answers = high score		274	(88.7)
20–23 correct answers		25	(8.0)
16–19 correct answers		249	(80.6)
12–15 correct answers		35	(11.4)
**Attitude domain**		**Yes, n**	**(%)**
24. Would you be willing to volunteer at a health facility during the COVID-19 outbreak?		130	(42.1)
25. Do you think that your health facility is prepared for a COVID-19 outbreak?		90	(29.1)
26. Do you agree that COVID-19 will be controlled in Ecuador?		71	(23.0)
27. Do you think that you are a potential source of contagion for your family?		303	(98.1)
**Practice domain**			
28. Is your health facility following a protocol or guideline to control COVID-19?		174	(56.3)
29. Do you have all the necessary safeguards and personal protective equipment for the care of COVID-19 patients delivered by your health facility?		29	(9.4)
30. Are you having conferences or talks/trainings on COVID-19 (diagnosis, handling of samples and biosecurity) in your health facility?		157	(50.8)
31. Since the start of the pandemic, do you do proper hand washing more often?		306	(99.0)
32. How often are you actively looking for information to stay informed about the SARS-CoV-2 pandemic?, n (%)	Daily	120	(38.8)
	Three times a week	79	(25.6)
	Twice a week	63	(20.4)
	Once a week	41	(13.3)
	It doesn’t	6	(1.9)
33. Most of the information you get about COVID-19 is from:, n (%)	Scientific articles	239	(77.3)
	Videoconferences	10	(3.2)
	News	49	(15.9)
	Social media	11	(3.4)

### Attitudes toward COVID-19

More than one-half of respondents had a negative attitude toward volunteering at a health facility during the COVID-19 outbreak (57.9%), and a majority of students reported that they did not have confidence that Ecuadorian health facilities and Ecuador, in general, could win the battle against COVID-19 (70.9% and 77.0%, respectively). The vast majority of respondents reported believing that they are a potential source of contagion for their families (98.1%). The univariate and multivariate analysis showed that not having personal protective equipment was associated with lack of willingness to volunteer in a health facility during the COVID outbreak (OR 4.07, *p* < 0·01); that male sex (OR 0.55, *p* = 0.03), not having personal protective equipment (OR 2.92, *p* = 0.01), and not having trainings (OR 3.07, *p* < 0.01), were associated with the feeling of the health facility not being prepared. Further, not having personal protective equipment was associated with the feelings that Ecuador will not control the outbreak (OR 2.70, *p* = 0.03) (Tables 3 and 4).

**Table 3:** Attitude’s questions towards COVID-19 by significantly associated variables

Variable	24. You be willing to volunteer					25. Your health facility is prepared for a COVID-19 outbreak					26. Ecuador will controlled COVID-19 outbreak				
Total participant *n* = 309	No, n (%)		Yes, n (%)		*p*-value	No, n (%)		Yes, n (%)		*p*-value	No, n (%)		Yes, n (%)		*p*-value
**Demographic variables**															
Sex										0·028					
Female	··	··	··	··	··	132	(42.7)	42	(13.6)	··	··	··	··	··	··
Male	··	··	··	··	··	87	(28.2)	48	(15.5)	··	··	··	··	··	··
**COVID-19 knowledge score**															
High	··	··	··	··	··	··	··	··	··	··	··	··	··	··	··
**Practice’s questions**															
29. Do you have personal protective equipment?					0·002					< 0·001					0·044
Yes	9	(2.9)	20	(6.5)	··	12	(3.9)	17	(5.5)	··	18	(5.8)	11	(3.6)	··
No	170	(55.0)	110	(35.6)	··	207	(67.0)	73	(23.6)	··	220	(71.2)	60	(19.4)	··
30 Are you having trainings?										< 0·001					
Yes	··	··	··	··	··	94	(30·4)	63	(20.4)	··	··	··	··	··	··
No	··	··	··	··	··	125	(40.5)	27	(8.7)	··	··	··	··	··	··

..: non-significant

**Table 4:** Multiple binary logistic regression analysis on factor significantly associated with attitude’s questions towards COVID-19

Variable	24. You are not willing to volunteer (vs. are willing)			25. Your health facility is not prepared for a COVID-19 outbreak (vs. is prepared)			26. Ecuador will not controlled COVID-19 outbreak (vs. will control)		
Total participant *n* = 309	OR	(95% CI)	*p*-value	OR	(95% CI)	*p*-value	OR	(95% CI)	*p*-value
**Demographic characteristic**									
Sex (male vs. female)	··	··	··	0.55	(0·32, 0·95)	0.031	··	··	··
**COVID-19 knowledge score**									
High vs. low	··	··	··	··	··	··	··	··	··
**Practice’s questions**									
29. Do you have personal protective equipment? (no vs. yes)	4.07	(1.65, 10.00)	0.002	2.92	(1.22, 6.90)	0.015	2.70	(1.11, 6.52)	0.029
30. Are you having trainings? (no vs. yes)	··	··	··	3.07	(1.75, 5.39)	< 0.001	··	··	··

..: non-significant

### Practices related to COVID-19

Only 50.8% of students reported they had any formal talks/training related to infection control, while 56.3% said their health facilities were following a guideline to control COVID-19. A small number of students reported they received all the necessary safeguards and personal protective equipment (9.4%). Nearly all students said they performed proper handwashing frequently (99.0%). A majority of respondents reported that they obtained information on COVID-19 from reliable sources (77.3% from scientific articles and 3.2% from videoconferences) (Table 2).

## Discussion

COVID-19 caused a global pandemic that has affected entire populations, from all over the world. The spread of SARS-CoV-2 has upended medical education and its importance within health systems. Owing to extensive hesitation and disagreement about the suitable roles for medical students during a pandemic, final-year medical student’s participation in clinical care has varied across nations [7]. In Ecuador, some medical schools forbid any patient interaction during the COVID-19 pandemic, whereas others have prepared their medical students for hospital-based roles, including final-year students currently serving as frontline healthcare workers [20, 21].

Medical students can enhance the efficiency of lightly staffed hospitals by taking medical related information, obtaining patient’s laboratory test, providing assistance to physicians and many other activities highly needed during a never-seen health crisis [7]. Although the main concern during a pandemic is to provide health services to reduce mortality, avoiding collateral infections within healthcare workers is mandatory. In our results, we found that the majority of final-year medical students had a high level of knowledge about COVID-19. The students were able to demonstrate their knowledge in viral transmission, and most of them have competence in how to manage COVID-19 patients. However, attitudes about the outlook of the disease and the willingness to volunteer during the COVID-19 outbreak in Ecuador were largely negative. Furthermore, we found evidence that the delivery of adequate personal protective equipment and implementation of training was poor.

To the best of our knowledge, this is the first study in Latin America examining knowledge, attitudes and practices towards diagnosis, treatment, prevention, prognosis, and education of COVID-19 among final-year medical students. The high levels of knowledge observed in our study are consistent with findings from recent surveys of HCWs in other areas of the world [16, 22–26]. However, it is difficult to directly compare our findings with those of previous studies because of varying definitions for establishing a “high” level of knowledge. This high score could be attributed to the severity of the pandemic and the overwhelming volume of articles, online conferences, and news reports related to this public health emergency, as found in other study [26]. However, a high score in theoretical knowledge is not sine qua non of good medical practice, because there are elements related to clinical practice and patient care that such evaluations cannot measure. For that reason, appropriate training is urgently necessary. Unfortunately, this need appears to be unmet by Ecuadorian health facilities and universities, given that almost half of final-year medical students in our study sample reported a lack of formal training about COVID-19. Although we do not have direct evidence of the magnitude of training deficits in other countries, the similarities in medical education models throughout Latin America make it reasonable to assume that lack of COVID-19 training may be prevalent among other countries in the region. This represents a concerning flaw and an important missed opportunity in the global fight against COVID-19. Future studies should assess these deficiencies in more detail and expand the scope to other Latin American countries to identify and improve on such training gaps.

Denmark and Mexico added the use of a novel digital platform to its undergraduate medical curriculum as a response to COVID-19. Evidence from its use suggested that timely changes to medical education can improve national responses to the COVID-19 pandemic [27, 28]. Such experiences demonstrate that Ecuadorian medical students may also benefit from additional opportunities to learn about COVID-19 management during this crisis. Findings from our study suggest that Ecuadorian final-year medical students, despite being the future of Ecuador’s front line of health care personnel, will not be adequately prepared to face the challenges of COVID-19 in daily clinical practice. This is particularly important because medical students are part of the solution to cover and complement the shortage of HCWs that exists in Ecuador. According to the latest data reported by INEC in 2018 [29], there are 23 doctors and 14 nurses per 10,000 inhabitants, that is equal and less than the minimum established by the WHO to provide essential health services to the population (23 skilled HCWs per 10,000) [30]. The number of these “new doctors” (3000) added to the existing doctors in Ecuador (39, 908) [29], would raise the number of doctors per 10,000 inhabitants to 25.2, higher than the minimum established by the WHO. To safely and effectively expand the HCW workforce, the development of a new curriculum is important; this will provide additional training to medical students equipping them to cover nursing, telemedicine, and social education tasks. This is predicted to have a positive impact as demonstrated in other countries where medical students were requested to collaborate in the triage and/or management of suspected or confirmed COVID-19 patients to avoid the collapse of the health systems [31, 32].

Similar studies on influenza and other infectious diseases assessing KAP in Latin American medical students demonstrated that students had a high level of knowledge of the disease and were pessimistic about possible government actions [33]. Our findings showing a high level of COVID-19 knowledge in Ecuadorian final-year medical students mixed with a pessimistic and negative attitude towards governments are understandable. These results contract with the results of other study carried out in a country with similar social and economic situation of Ecuador [22]. Perhaps the lack of adherence to social distancing measures, the lack of isolation of positive cases (which is difficult in a country where cultural and economic inequalities have led people from informal jobs or underemployment not to follow the recommendations), decades of poor political management of the health sector, and poor management of the country’s medical and student organizations are related to this pessimistic vision of the pandemic’s future [34]. Furthermore, the absence of official and unambiguous statements on indemnity, expected roles and responsibilities, and contractual agreements would make it difficult for students to participate in Ecuador. Comparable issues have been reported in other countries, where they suggest that poor government strategies have negatively impacted on medical education [22]. Additionally, these students’ negative attitudes could result from the shortage of protective equipment and medical supplies, considering that 90.6% of students said they do not have appropriate personal protective equipment.

We hope that this study’s results encourage the development of public health policies that improve the decision-making process against a disease without borders and promote and inform the creation of an evidence-based national training curriculum against COVID-19 (aimed at preparing and educating medical students). Moreover, we believe that having an adequate level of knowledge of COVID-19 will allow this type of program to be implemented efficiently, which will be of great benefit during this pandemic and for the future.

### Limitations

Our study has several limitations. The main limitation of this study is that it can only generate an overview of what is happening with final-year medical students, as we did not reach a 100% response rate, which did not allow us to present conclusive results. However, the response rate should not be the only consideration when judging survey quality and bias as sometimes it is impossible to make the subjects respond. It can be related to factors like; survey fatigue (as many survey-based studies were carried out during that time), stress and anxiety about COVID-19, and lack of incentive. The short period of time for conducting the survey may also contribute to the response rate, nevertheless, this was necessary to assess knowledge gaps during the critical initial phase of the pandemic, in order to evaluate how prepared final-year medical students were to respond to this public health emergency. Second, the non-random sampling of this study could be a source of self-selection bias. Although it is not possible to predict this bias’s direction, we believe that it is reasonable to expect that knowledge could be overestimated if those with lower knowledge chose not to participate. Similarly, those who were more optimistic about the situation may have been more inclined to participate, which could have underestimated negative attitudes and inadequate behavioural patterns. Third, since the survey was also sent by social media a sample selection bias could occurs. We try to manage it using groups related to final-year medical students. Future studies could address this limitation by selecting a random sample of medical students, perhaps through institutional e-mails. Social desirability bias is another potential limitation that could have affected attitudes and behavioural pattern responses due to the self-report nature of the survey. In other words, negative attitudes and inadequate behavioural patterns may be underestimated due to respondents having a desire to mark what they consider to be “socially acceptable” responses. However, the use of an anonymous online survey should have mitigated the risk of this bias. Future studies could also avoid this bias by implementing direct observation of practice to get more accurate behavioural patterns estimates. Despite these limitations, we consider this study to be a reliable approximation of knowledge, attitudes, and practice of Ecuadorian medical students, as this study is the first in Ecuador and Latin America, and can help to inform their training needs and, subsequently, could be used to design a specific curriculum about COVID-19 that could act as an essential healthcare resource.

## Conclusions

Like many middle-income countries, Ecuador does not have operational readiness capacities or a definitive and actionable plan to combat a pandemic at the scale of COVID-19. The high level of knowledge of final-year medical students is an essential resource that Ecuador will soon tap into. Despite the majority being pessimistic about possible government actions on the public health system, a non-depreciable percentage of students were willing to volunteer, therefore it is imperative to have access to protective equipment to ensure their safety. Ecuador’s education system must strengthen students’ training while considering their personal development and attitudes. Creating a national curriculum that considers public health guidelines for students’ education will prepare them to efficiently contribute to population health maintenance when facing emergency health situations like COVID-19 while addressing their concerns that were raised in this study.

## Supplementary Information

**Additional file 1.**

## Data Availability

The dataset with the total of 309 responses can be obtained from the authors upon reasonable request. The questionnaire summary and its results are included in this article.

## Abbreviations

SARS-CoV-2: The severe acute respiratory syndrome coronavirus-2
COVID-19: The novel coronavirus disease-2019
HCWs: Healthcare workers
AFEME: Association of Ecuadorian Faculties of Medical and Health Sciences
WHO: The World Health Organization?
INEC: National Institute of Statistics from Ecuador
