# Supplementary material for: Paradigms about the COVID-19 pandemic: knowledge, attitudes and practices from medical students
Source: BMC Med Educ. 2021 Feb 24;21:128. doi: 10.1186/s12909-021-02559-1 (PMC7903404; doi:10.1186/s12909-021-02559-1)
Supplement: Supplementary file 1 — Additional file 1. [file 12909_2021_2559_MOESM1_ESM.pdf]

Paradigms about the COVID-19 pandemic: knowledge, attitudes and practices from medical students

Lincango-Naranjo Eddy, MD[1,2], Espinoza-Suarez Nataly, MD[1], Solis-Pazmino Paola, MD[3], Vinueza-Moreano Paul, MD[2], Rodriguez-Villafuerte Santiago, PhD[4,5], Lincango-Naranjo Jose[6], Barberis-Barcia Giuseppe, MD[2], Ruiz-Sosa Carlos, MD[2] , Rojas-Velasco Giovanni, PhD[2,8], Gravholt Derek[1], Golembiewski, Elizabeth, PhD[1], Soto-Becerra Percy, MD[8], Maryam Khan[9] and Esteban Ortiz-Prado[10]

[1] Knowledge and Evaluation Research Unit, Mayo Clinic, Rochester, Minnesota, 55905, USA.

[2] Facultad de Ciencias Médicas, Universidad Central del Ecuador, Quito-Ecuador.

[3] Department of Otolaryngology-Head and Neck Surgery, School of Medicine, Stanford University, Stanford, California, USA.

[4] Facultad de Medicina, Programa de Pós-graduação em Hepatología, Universidade Federal de Ciências da Saúde de Porto Alegre (UFCSPA), Porto Alegre, RS, Brasil.

[5] Hospital Vozandes Quito, Quito, Ecuador.

[6] Universidad de las Fuerzas Armadas, Quito, Ecuador.

[7] Universidad de Sao Paulo.

[8] Centro de Excelencia en Investigaciones Económicas y Sociales de Salud, Universidad San Ignacio de Loyola, Lima, Perú

[9] University Hospital Southampton

[10] OneHealth Global Research Group, Universidad de las Americas, Quito, Ecuador

Corresponding author:

*Corresponding author: Esteban Ortiz-Prado One Health Research Group, Universidad de las Américas, Quito, Ecuador Calle de los Colimes y Avenida De los Granados, Quito 170137, Ecuador. Email: e.ortizprado@gmail.com Phone: +593995760693

Survey questions and answer options

Participation agreement form: Do you want to voluntarily participate in the survey and give your consent to use your anonymous data for research?

Yes/No

Demographic characteristics

1. Sex
   Female, Male.
2. Age
3. Study city
   Quito, Guayaquil, Cuenca, Ambato, Manta, Esmeraldas, Tulcan, Ibarra, Riobamba, Latacunga, Loja, Machala, Quevedo, Santa Elena.
4. University
   Public, Private.
5. Hospital type
   Public, Private.
6. Department
   Emergency, Intensive Care Unit, Respiratory, Internal Medicine, Gynecology, Pediatrics, others (specify).

Knowledge domain

General knowledge

1. Is SARS-CoV-2 a new coronavirus identified at the end of 2019, and when it infects humans, causes acute respiratory infection?
   True, False, Not sure.
2. Is SARS-CoV-2 a positive-sense single-stranded RNA virus?
   True, False, Not sure.
3. Is SARS-CoV-2 a member of the subgenus Sarbecovirus (beta-CoV B lineage)?
   True, False, Not sure.
4. Can SARS-CoV-2 live for some time on some surfaces?
   True, False, Not sure.
5. Not everyone with COVID-2019 will develop serious cases. Are people who have the following characteristics more likely to have serious cases? : age over 65 years, have comorbidities, obese.
   True, False, Not sure.
6. Would being in contact with your pets lead to SARS-CoV-2 virus infection?
   True, False, Not sure.
7. Do you think SARS-CoV 2 stays in the air for 3 hours?
   True, False, Not sure.

Diagnosis

8. Are the most prevalent symptoms of COVID-19 fever, cough and fatigue?
   True, False, Not sure.
9. Is the main incubation period for COVID-19 1-30 days?
   True, False, Not sure.
10. Is the main transmission mechanism of SARS-CoV-2 close person-to-person contact between people infected with the virus, whether symptomatic or asymptomatic?
    True, False, Not sure.
11. Is the diagnosis for COVID-19 recommended by the WHO made by a rapid test?
    True, False, Not sure.
12. Is the diagnosis for COVID-19 recommended by the WHO made by the polymerase chain reaction (RT-PCR) test of the nasopharyngeal swab?
    True, False, Not sure.
13. Is a suspicious case of COVID-19 defined as any patient who meets the clinical picture of acute respiratory syndrome (fever, cough, dyspnea, fatigue), and / or epidemiological criteria (being in contact with a suspected or confirmed case of COVID 19, travel or residence in area with active infections in the last 14 days), but without confirmation by laboratory test?
    True, False, Not sure.
14. Is a confirmed case of COVID-19 defined as any patient who meets the clinical picture of acute respiratory syndrome and / or epidemiological criteria confirmed by laboratory testing?

Treatment

15. There is currently no effective cure for COVID-2019, but does early supportive and symptomatic treatment help most patients recover from infection?
    True, False, Not sure.
16. Is the use of chloroquine is recommended as prophylaxis for COVID-2019?
    True, False, Not sure.

Prevention

17. Should hand hygiene be performed for more than 20 seconds, mainly hand washing with soap?
    True, False, Not sure.
18. Does the personal protective equipment recommended by the WHO, for the care of a suspected or confirmed case of COVID-19, without aerosol-generating procedures, include: hand hygiene, N95 mask, gloves, gown, and protective glasses?
    True, False, Not sure.
19. Was the recommendation of distance between patients and health personnel as far as possible due to the fact that the macroparticles generated by coughs or sneezes spread up to 2 meters away, and therefore are potential virus transporters?
    True, False, Not sure.
20. Do you think that the life time of the N95 mask is 7 days?
    True, False, Not sure.
21. Is Isolation an effective way to reduce the spread of the virus?
    True, False, Not sure.

Prognosis

22. Can patients affected by COVID-19 recover from the disease?
    True, False, Not sure.
23. Can people recovered from the disease still transfer or spread it?
    True, False, Not sure.

Attitude domain

24. Would you be willing to volunteer at a health facility during the COVID-19 outbreak?
    Yes, No.
25. Do you think that your health facility is prepared for a COVID-19 outbreak?
    Yes, No.
26. Do you agree that COVID-19 will be controlled in Ecuador?
    Yes, No.
27. Do you think that you are a potential source of contagion for your family?
    Yes, No.

Practice domain

28. Is your health facility following a protocol or guideline to control COVID-19?
    Yes, No.
29. Do you have all the necessary safeguards and personal protective equipment for the care of COVID-19 patients delivered by your health facility?
    Yes, No.
30. Are you having conferences or talks/trainings on COVID-19 (diagnosis, handling of samples and biosecurity) in your health facility?
    Yes, No.
31. Since the start of the pandemic, do you do proper hand washing more often?
    Yes, No.
32. How often are you actively looking for information to stay informed about the SARS-CoV-2 pandemic?
    Daily, Three times a week, Twice a week, Once a week, It does not
33. Most of the information you get about COVID-19 is from:
    Scientific articles, Videoconferences, News, Social media
